# Pre-existing systemic inflammation attenuates bacterial clearance by the liver in porcine abdominal sepsis

**DOI:** 10.1186/2197-425X-3-S1-A620

**Published:** 2015-10-01

**Authors:** K Hanslin, J Sjölin, P Skorup, F Wilske, R Frithiof, A Larsson, M Castegren, M Lipcsey

**Affiliations:** Department of Medical Sciences, Uppsala University, Section of Infectious Diseases, Uppsala, Sweden; Department of Surgical Sciences, Uppsala University, Section of Anesthesiology and Intensive Care, Uppsala, Sweden; Department of Medical Sciences, Uppsala University, Section of Clinical Chemistry, Uppsala, Sweden

## Introduction

Bacterial translocation from the gut has been suggested to induce and maintain the systemic inflammatory response (SIRS) and organ dysfunction. The reticulo-endothelial system in the liver has a pivotal role in clearing the blood from circulating bacteria. To date, the effects of SIRS on hepatic bacterial clearance are not fully understood.

## Objectives

To investigate if pre-existing inflammation, as seen in sepsis, influences the trans-hepatic bacterial clearance.

## Methods

The study was conducted on anesthetized pigs in an intensive care setting. All animals were subjected to an infusion of *E coli* in the portal vein for 3 hours. In Group 1 (n=6), a systemic inflammatory response was induced by a continuous intravenous endotoxin infusion starting 24 h prior to the bacterial infusion. Group 2 (n=6) received the bacterial infusion without prior endotoxin exposure. Three animals (Group 3), serving as controls for effects of 24 h anesthesia, received saline instead of endotoxin for 24 h prior to the bacterial infusion. Bacterial counts in the portal and hepatic vein, as well as the levels of tumor necrosis factor-α (TNF-α) in venous blood were analyzed hourly during the bacterial infusion.

## Results

**Figure 1:**
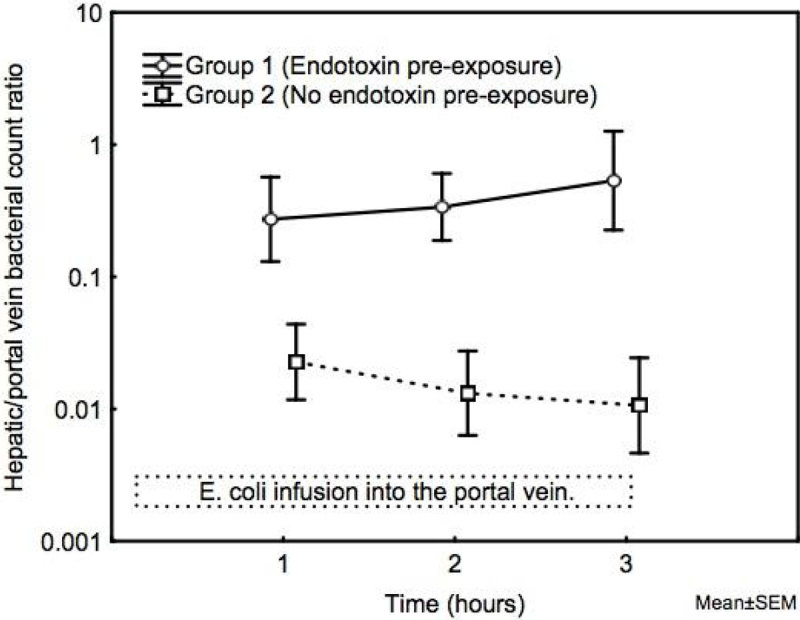
Figure 1

All animals subjected to endotoxin developed SIRS. The amounts of bacteria administered were comparable between the groups. The ratio of hepatic to portal vein bacterial counts was higher in Group 1 vs. 2 (p < 0.001; Figure 1). Peak plasma levels of TNF-α in Group 1 (1957 pg/mL (1843-2092) median (IQR)) were lower compared to Group 2 (69200 pg/mL (60305-77350); p < 0.001). Group 3 was numerically intermediate in bacterial counts and TNF-α levels and, according to the predefined plan, not included in the primary analysis.

## Conclusions

Our results suggest that despite lower inflammatory response to bacterial infusion, the trans-hepatic bacterial clearance is a magnitude lower in pigs with pre-existing systemic inflammatory response. Should similar mechanisms operate in human critical illness, then hepatic clearance of bacteria from the gut is impaired by SIRS.

